# The NCD/COVID-19 intimidating relationship: An urgent call for countries in the WHO Eastern Mediterranean Region

**DOI:** 10.7189/jogh.11.03010

**Published:** 2021-01-16

**Authors:** Saverio Bellizzi, Gabriele Farina, Luca Cegolon, Giuseppe Pichierri, Catello M Panu Napodano, Alessio Santoro, Dina Sabry Said, Yehia Alzoubi

**Affiliations:** 1 Medical Epidemiologist, Independent Consultant, Geneva, Switzerland; 2 University of Sassari, Sassari, Italy; 3 Local Health Unit N.2 “Marca Trevigiana”, Public Health Department, Treviso, Italy; 4 Kingston Hospital NHS Foundation Trust, Microbiology Unit, Kingston Upon Thames, UK; 5 Public Health Specialist, Independent Consultant, Milan, Italy; 6 College of Business Administration, American University of the Middle East, Kuwait

A modeling study published in the second semester of 2020 has suggested how two out of 10 people globally are at increased risk of severe COVID-19 mostly because of underlying non-communicable diseases (NCD) [1]. At the same time, the gigantic efforts to face the COVID-19 pandemic have led to the disruption of regular care provision services in more than one/third of all countries in the world [2].

Some striking examples of the effect of underlying NCD and disruption of NCD services on morbidity and mortality due to COVID-19 include Italy where among those dying of COVID-19 in hospitals, 68% had hypertension and 31% had type-2 diabetes; similarly, in India 30% fewer acute cardiac emergencies reached health facilities in rural areas in March 2020 compared to the previous year, and in the Netherlands the number of people newly diagnosed with cancer dropped by 25% as a result of the lockdown [3].

The WHO Eastern Mediterranean Region (EMR) has one of the highest prevalence of NCDs globally. Given an adult hypertension prevalence between 20% and 30% and a diabetes prevalence of 14%, NCDs represent one of the greatest health burdens in the Region [4]. This translates into more than 100 million people living with hypertension [5], 50 million people with diabetes [6], and 1.35 million with cancer [7].

On the other hand, more than 1.7 million people in the EMR die every year from cardiovascular diseases, cancer, chronic respiratory diseases and diabetes; the contribution in EMR of these four main NCDs is estimated to raise the respective attributable mortality to 2.4 million deaths by 2025 unless action is taken [8].

We must consider that the Region also hosts the majority of the world’s current refugees and internally displaced persons (IDPs) [4], who are more and more in need of NCDs assistance: the epidemiological transition from communicable to non-communicable diseases has for instance obliged the International Committee of the Red Cross (ICRC) over time to re-orient its medical response in countries like Lebanon, Pakistan, Sudan, Syria and Iraq, where diabetes contributes to more than one fourth of amputations among 30% of the centres in the cohort of amputees within ICRC’s Physical Rehabilitation Centres [9].

**Figure:**
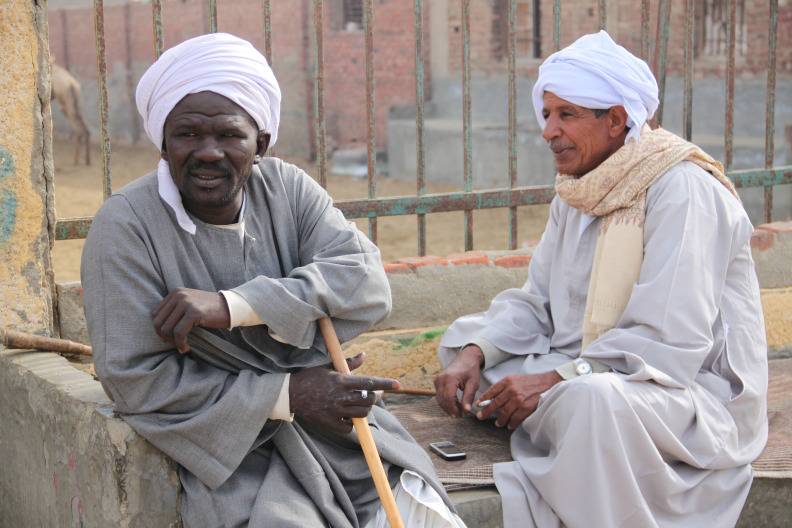
Photo: This photo, taken in the countryside of the Nile delta shows two men smoking cigarettes, which is one of the major contributors of NCD in the region (from the author’s collection, used with permission).

The significant surge of NCDs in the EMR over recent years has a multifactorial origin. However, the main determinants driving this health shift are clear and are related to tobacco use, poor diet, and physical inactivity: 90 million adults in EMR are tobacco users while 185 million are overweight or obese and 130 are physically inactive.

Smoking, which is associated with increased severity of disease and death in hospitalized COVID-19 patients [10], represents a huge issue: compared to a high probability of a decline in smoking prevalence for most countries in other WHO Regions, the prevalence in the EMR is likely to increase, especially among males [11]. Slow pace of implementation of tobacco control measures in several countries is critical and will most likely hinder attempts to progress universal health coverage goals [11].

Overweight and obesity, whose mechanism underlying the increased risk of mortality from COVID-19 is still poorly understood, is of particular concern in countries such as Egypt, Bahrain, Jordan, Kuwait, Saudi Arabia and United Arab Emirates, where its prevalence ranges from 74% to 86% in women and 69% to 77% in men [12].

As highlighted in the joint statement of the EMT NCD Alliance during the recent 67th WHO Regional Committee for EMRO [13], Member States need to integrate NCD prevention and control into COVID-19 preparedness and response plans. This would require health promotion interventions in the three main settings recommended by WHO: schools, workplaces (as this is considered as a non-work hindrance stressor that may affect employees’ performance and productivity in the workplace) and primary health care settings. The implementation of health registries would enable to monitor the health of the general population and evaluate any public health intervention. The involvement of civil society and affected communities, including those in humanitarian settings, is key for decision-making and monitoring processes. Finally, ensuring a people-centered and inclusive approach as well as the availability of NCD medicines, supplies, and care alongside those for communicable diseases like COVID-19 is of paramount importance towards Universal Health Coverage.
